# Hyperbilirubinemia and phototherapy differentially alter hippocampal transcriptome in the preterm Gunn rat model

**DOI:** 10.1038/s41390-025-04700-y

**Published:** 2025-12-22

**Authors:** Katherine M. Satrom, Eric F. Lock, Troy C. Lund, Phu V. Tran, Raghavendra B. Rao

**Affiliations:** 1 Division of Neonatology, Department of Pediatrics, University of Minnesota, Minneapolis, MN USA; 2 Division of Biostatistics and Health Data Science, School of Public Health, University of Minnesota, Minneapolis, MN USA; 3 Division of Blood and Marrow Transplant, Department of Pediatrics, University of Minnesota, Minneapolis, MN USA

## Abstract

**Background:**

Preterm infants are at risk for bilirubin-induced brain injury. Phototherapy is effective for lowering serum bilirubin but has potential adverse effects. The independent effects of hyperbilirubinemia and phototherapy on the hippocampal gene expression profile were determined using a preterm-equivalent Gunn rat model.

**Methods:**

Jaundiced and non-jaundiced pups were subjected to phototherapy from postnatal day (P) 4 through P6. Hippocampal transcriptome was assessed on P6 using genome-wide RNA sequencing (RNAseq) followed by qPCR validation of top 5 upregulated and 5 downregulated (>1.8- absolute fold change) genes.

**Results:**

Phototherapy lowered serum bilirubin in jaundiced pups on P6, compared with untreated pups (5.37 ± 0.54 mg/dL vs. 8.83 ± 0.55 mg/dL, *p* < 0.0001). RNAseq identified 1294 differentially expressed genes (DEG) for hyperbilirubinemia, 3297 DEGs for phototherapy, with 407 overlap DEGs. qPCR confirmed the expression of all top upregulated and downregulated genes affected by phototherapy (*Ano3, Gabarapl2, Myo16, Vsnl1, Arhgef9, Rnfl6, Xpo5, Mcm3, Draxin*) except *Dnmt1*.

**Conclusion:**

Both hyperbilirubinemia and phototherapy altered the transcriptome of the developing rat hippocampus with phototherapy having a 2.5-fold greater impact than hyperbilirubinemia. The top transcripts identified indicate that phototherapy impacts important CNS functions including neurogenesis, synaptogenesis, and microtubule dynamics.

**Impact:**

- This study utilized a model of phototherapy treatment in a preterm Gunn rat model of hyperbilirubinemia.
- Data demonstrate that mild hyperbilirubinemia, and to an even greater extent, phototherapy, induces widespread gene expression changes in the developing hippocampus.
- Phototherapy was associated with differentially expressed genes related to neurogenesis, synaptogenesis, and microtubule dynamics.

## Introduction

Unconjugated hyperbilirubinemia is common soon after birth in newborn infants. High levels of unconjugated bilirubin (UCB) are neurotoxic and lead to neuronal injury, affecting the basal ganglia, cerebellum, auditory pathways, and the hippocampus.^1^ Preterm infants are at risk for bilirubin-induced brain injury at lower serum UCB levels.^2–4^

Phototherapy is effective for lowering UCB levels and is used commonly in the preterm population to prevent bilirubin neurotoxicity.^4–7^ However, the full range of effects of phototherapy on preterm infants are not well understood. In term infants, there is an increased risk of childhood seizures in infants treated with phototherapy.^8^ A clinical trial demonstrated that aggressive phototherapy to lower UCB levels is associated with an increased risk of death in extremely preterm infants.^9^ In contrast to its harmful effects, UCB potentially has a protective effect as an antioxidant.^10^ There is evidence in adult humans that UCB protects against inflammatory and oxidant-mediated diseases,^11–13^ and some types of cancer.^14,15^

Previous studies have demonstrated that phototherapy is associated with genotoxic and metabolomic changes in the serum of term^16,17^ and preterm infants.^7^ The effects of phototherapy on the brain regions targeted by UCB are not well understood. The objective of the present study was to determine the independent and combined effects of UCB and phototherapy on the hippocampal transcriptome in a preterm-equivalent Gunn rat model of unconjugated hyperbilirubinemia. Gunn rats have a mutation in UDP-glucuronyl transferase (UGT1), the enzyme responsible for conjugation of bilirubin in the liver and are used to model neonatal hyperbilirubinemia and bilirubin encephalopathy.^18–21^ Homozygous (*Ugt1a1*^*Gunn/Gunn*^; jj group) Gunn rat pups develop hyperbilirubinemia soon after birth that is evident by epidermal yellow discoloration (i.e., jaundice), while the heterozygous (*Ugt1a1*^*+/Gunn*^, Nj group) littermates have reduced activity of the UGT1 enzyme and remain jaundice-free. Similar to human neonates, hyperbilirubinemia responds to phototherapy in Gunn rats demonstrated in lowering serum and brain bilirubin levels.^22–25^ We focused on the hippocampus because preterm infants are at an overall increased risk for long-term hippocampal-mediated neurocognitive deficits,^26^ and it is one of the brain regions affected in bilirubin encephalopathy.^1^ Although there are studies that demonstrate transcriptomic effects of bilirubin on the developing brain regions^27–29^ including the hippocampus,^30,31^ to our knowledge, there are no studies evaluating the effects of phototherapy on the hippocampal transcriptome in a Gunn rat model.

## Materials and methods

### Animals

Experiments were performed with the approval of the Institutional Animal Care and Use Committee of the University of Minnesota. Pregnant dams were purchased (Rat Resource & Research Center, Columbia, MO; RRRC#341: Gunn-*Ugt1a1*^*j*^/BluHsdRrrc, RRID:RRRC 00341) and housed under standard laboratory conditions on an *ad lib* diet and 12 h:12 h light and dark cycles. Nj female and jj male matings were employed to produce a litter with approximately equal number of Nj and jj pups. Dams were allowed to deliver spontaneously, and pups were left undisturbed until P4 to promote bonding and avoid stress. Littermate Nj rat pups served as controls. Pups from a total of five litters were used. Both male and female pups were studied. Data were pooled as no sex-specific effects have been demonstrated in the neonatal Gunn rat model.^18^

### Phototherapy

**Fig. 1:**
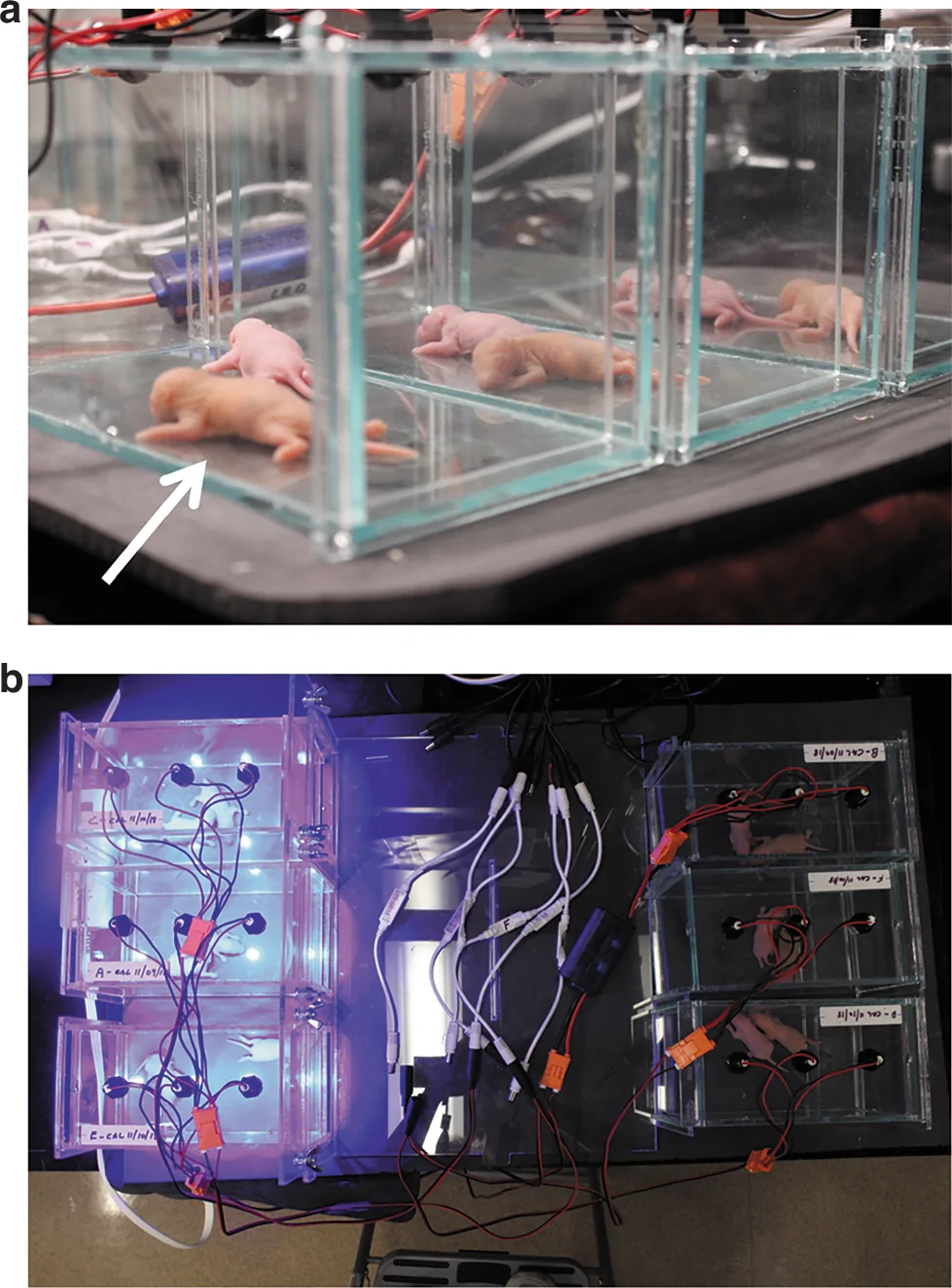
Experimental groups and the phototherapy apparatus. **a** Two Gunn rat pups: One heterozygous Gunn rat (Nj) and one homozygous Gunn rat (jj, white arrow) per chamber. **b** LED lights on in treatment groups (left) and LED lights OFF in control groups (right).

**Fig. 2:**
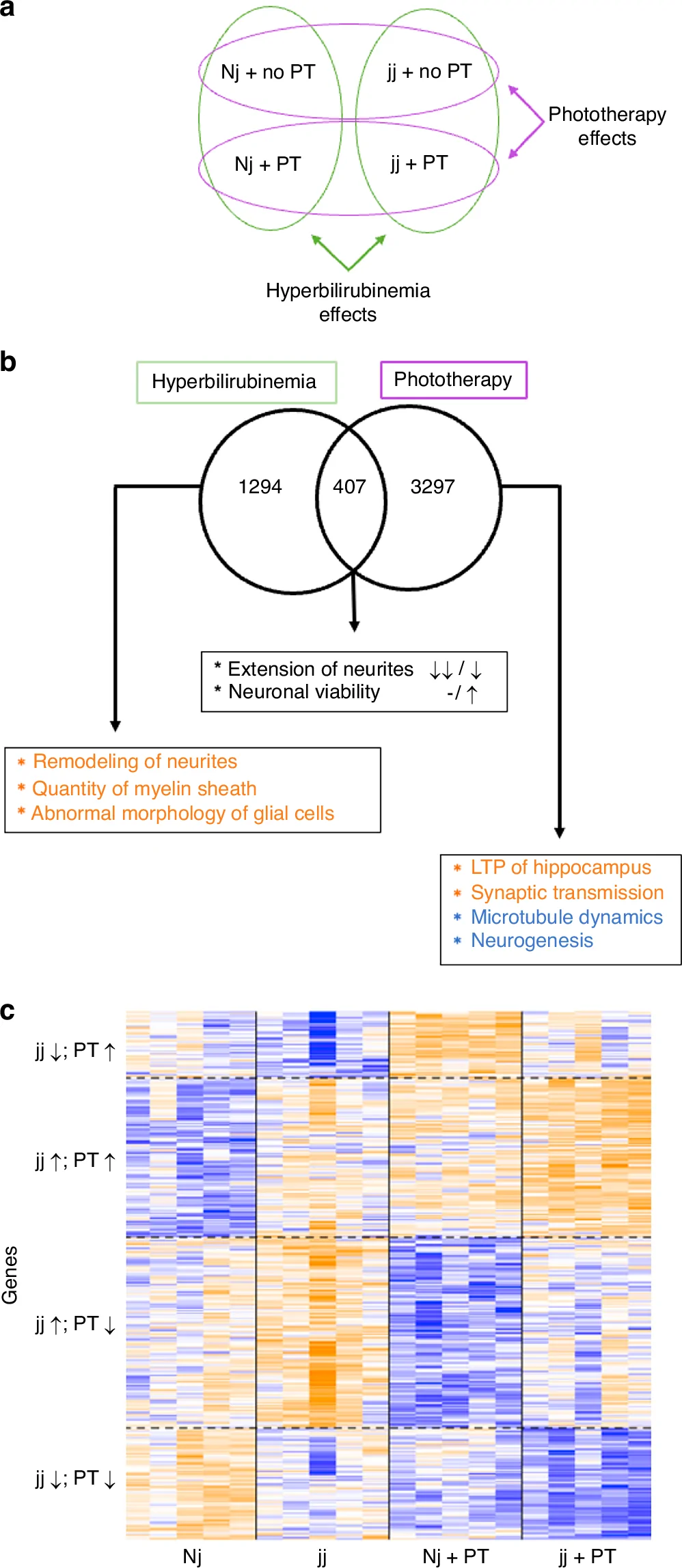
Four-group comparison and associated gene expression changes and biological functions. **a** Schematic of the four-group comparison for analysis highlighting what is represented in the hyperbilirubinemia effect vs. phototherapy effect comparison for gene expression changes. **b** Biological functions determined by IPA affected by hyperbilirubinemia, phototherapy, and by both conditions (middle of Venn diagram). **c** Heat Map of 407 overlap transcripts altered by both hyperbilirubinemia and phototherapy. The four treatment groups are represented on the x-axis. The arrows in the y-axis indicate whether gene expression is relatively higher (up arrow) or lower (down arrow) in the hyperbilirubinemia groups (‘jj’: jj+no PT and jj+PT) and phototherapy groups (‘PT’: Jj+PT and jj+PT). Blue indicates decreased transcript expression and orange indicates increased expression. LTP long term potentiation, Nj heterozygous Gunn rat pups, jj homozygous Gunn rat pups, No PT no phototherapy, PT phototherapy.

Both Nj and jj Gunn rat pups (*n* = 6–8/group) were exposed to intermittent LED phototherapy using a custom-built phototherapy device (Fig. 1, Arbor Grace Inc, Detroit, MI) from P4 through P6. These time points were chosen because they neurodevelopmentally approximate the brain of human extremely preterm infants.^32–35^ The phototherapy device was comprised of 6 individual chambers that allowed simultaneous delivery of phototherapy to pairs of pups (Fig. 1b). Body temperature was maintained using heating pads placed under the chamber. The irradiance of the LED lights was 60 μW/cm^2^/nm at the surface of the pups as measured by a light meter (Ohmeda Biliblanket® Light Meter II; GE Healthcare, Maple Grove, MN). Phototherapy was provided for a total of 12 h (3 h on/3 h off during daytime hours allowing for time with dams and feeding during time off treatment). To ensure that maternal separation by itself did not affect bilirubin levels or body weights, a separate set of Nj and jj pups (*n* = 6–8/group) were placed in the chamber with phototherapy lights OFF for an equivalent duration of time from P4 to P6. Identical laboratory conditions, including exposure to ambient light, humidity, and animal handling were used in both experiments. Untreated Nj and jj pups (*n* = 6–8) were used as controls to determine the independent effects of hyperbilirubinemia, and both heterozygous (Nj) and homozygous (jj) pups exposed and unexposed to phototherapy were compared (Fig. 2a).

### Blood and hippocampal tissue isolation and bilirubin measurement

Rats were killed on P6 using an injection of sodium pentobarbital (100 mg/kg, i.p.). A blood sample (250 µL) was collected via cardiac puncture. Hippocampi were dissected on an ice-cold glass block, flash-frozen using liquid nitrogen, and stored at −80 °C until analysis.

Total serum bilirubin (TSB) levels were determined on P6 using the UNISTAT® Bilirubinometer (Reichert Technologies, Depew, NY) and reported as mean ± SEM.

### RNA sequencing

Total RNA was isolated from one hippocampus using RNAqueous Isolation kit (Ambion) as in our previous study.^36^ Five rats were used per experimental group (Nj+no phototherapy (PT), jj+no PT, Nj+PT, jj+PT). RNA was quantified using RiboGreen RNA Assay kit (Invitrogen) and quality assessed with capillary electrophoresis (Agilent BioAnalyzer 2100; Agilent). Libraries were created for each sample using TruSeq stranded mRNA kit (Illumina) and were size selected for ~200 bp inserts. Sequencing was completed with Illumina HiSeq 2500 to generate 50-bp paired-end reads. Sequencing depth was on average >30 M reads per sample.

### Bioinformatics and pathway analysis

Quality control for the raw sequenced reads was performed with FastQC. All samples had an average quality score of 36. Read mapping was performed via Hisat2 (v2.1.0) using the rat genome (Rnor_6.0.98) as reference. Gene quantification was performed with Feature Counts for raw read counts. Differentially-expressed genes (DEGs) between groups were identified using the EdgeR (negative binomial) feature in CLCGWB (Qiagen, Redwood City, CA) using raw read counts and *p* < 0.05. DEGs were analyzed using the knowledge-based database Ingenuity Pathway Analysis (IPA; Qiagen), which consists of more than 7 million findings, to identify biological processes and canonical pathways altered by hyperbilirubinemia and phototherapy, as previously described.^36,37^ The search criteria included indirect and direct interactions, mammalian tissues, and nervous system. Significance was set at absolute z-score ≥ 2.0.

### Quantitative Real-Time Polymerase Chain Reaction (qPCR)

Hippocampal mRNA concentration was quantified for the top five upregulated and top five downregulated DEGs identified with a phototherapy effect by RNAseq. Commercial gene expression assays (Taqman Gene Expression Assays, Applied Biosystems, Carlsbad, CA; Supplementary Table S1) were used as described in our previous studies.^38^ Samples from the four groups (*n* = 5–7/group) were assayed in duplicate using the cycle threshold (ΔΔCT) method and ribosomal protein *S18* as (normalizer) control.

### Statistical analysis

TSB levels among the four groups were compared using two-way ANOVA with Tukey’s multiple comparisons test. The independent and interdependent effects of genotype and phototherapy on hippocampal transcript expression were determined by ANOVA. All statistical analyses were performed using GraphPad Prism (La Jolla, CA). Statistical significance was set at alpha < 0.05.

## Results

### Phototherapy lowers serum bilirubin levels in jaundice (jj) pups

Hyperbilirubinemia was evident clinically within approximately 24 h after birth by yellow discoloration of the skin in the homozygous jj pups, but not in the heterozygous Nj littermates (Fig. 1a, arrow). On P6, mean TSB levels in the jj+PT group were significantly lower compared with the untreated jj Gunn rat pups (5.37 ± 0.54 mg/dL vs. 8.83 ± 0.55 mg/dL, *p* < 0.0001.) The Nj Gunn rat pups did not have hyperbilirubinemia, and phototherapy had no effect on TSB levels in this group (0.35 ± 0.22 mg/dL vs. 0.28 ± 0.17 mg/dL, *p* > 0.999, adjusted on multiple comparisons test). Change in body weight over the treatment period was no different between the pups exposed to phototherapy and those that were separated from the mothers but did not receive phototherapy.

### Hyperbilirubinemia and phototherapy differentially alter the hippocampal transcriptome

Within the ANOVA framework, the interaction tests whether the effect of phototherapy differs for jj vs. Nj rats. There was not a significant interaction effect after adjusting for multiple comparisons via the false discovery rate (FDR > 0.1). Thus, we focus on the main effects of phototherapy and hyperbilirubinemia, rather than specific group-wise comparisons (provided in Supplementary Table S2).

1701 DEGs were found between Nj and jj Gunn rat pups regardless of phototherapy exposure (Nj+no PT and Nj+PT vs. jj+no PT and jj+PT), representing an overall effect of hyperbilirubinemia on hippocampal gene expression. Phototherapy resulted in 3704 DEGs regardless of the presence or absence of hyperbilirubinemia (Nj+no PT and jj+no PT vs. Nj+PT and jj+PT), representing an overall effect of phototherapy on gene expression. A total of 407 DEGs were common between hyperbilirubinemia and phototherapy effects, representing gene expression changes that were altered by both conditions (Fig. 2b). Table 1 includes the top five upregulated and top five downregulated DEGs associated with phototherapy exposure. The full list of identified DEGs is provided in Supplementary Material.

**Table 1:** Top five upregulated and top five downregulated transcripts in phototherapy groups identified by RNA sequencing with relative hippocampal mRNA transcript expression by qPCR. qPCR values are represented as mean ± SD *n* = 5–7/group; Two-way ANOVA with Tukey’s multiple comparison test; Control group transcript expression is normalized to 1.00.

			RNA sequencing		qPCR validation (mean ± SD)						
Gene name	Gene Symbol	Function	Fold Change (Log_2_)	FDR	Nj	jj	Nj + PT	jj + PT	Jaundice p-value	PT p-value	Interaction p-value
anoctamin 3	*Ano3*	Plasma membrane component; calcium-mediated signaling	1.88	<0.01	1.00 ± 0.23	0.84 ± 0.14	1.73 ± 0.37	2.07 ± 0.50	0.53	<0.001	0.09
GABA type A receptor associated protein like 2	*Gabarapl2*	Autophagy, mitochondrial function	1.86	<0.001	1.00 ± 0.06	0.92 ± 0.03	1.12 ± 0.13	1.16 ± 0.14	0.67	<0.001	0.17
visinin-like 1	*Vsnl1*	CNS development, calcium-mediated signaling	1.85	<0.01	1.00 ± 0.08	1.00 ± 0.11	1.75 ± 0.18	1.75 0.28	0.98	<0.001	0.99
cdc42 guanine nucleotide exchange factor 9	*Arhgef9*	GABA-mediated synaptic transmission, postsynaptic specialization	1.83	<0.01	1.00 ± 0.08	1.06 ± 0.09	1.33 ± 0.14	1.56 ± 0.25	<0.05	<0.001	0.18
ring finger protein 6	*Rnf6*	Regulation of axon extension, enables DNA binding activity	1.82	<0.001	1.00 ± 0.08	0.85 ± 0.09	1.32 ± 0.10	1.39 ± 0.14	0.39	<0.001	<0.05
			**RNA sequencing**		**qPCR Validation (mean **± **SD)**						
**Gene name**	**Gene Symbol**	**Function**	**Fold Change**	**FDR**	**Nj**	**jj**	**Nj** + **PT**	**jj** + **PT**	**Jaundice p-value**	**PT p-value**	**Interaction p-value**
myosin XVI	*Myo16*	Actin filament binding activity	-1.83	<0.01	1.00 ± 0.06	1.06 ± 0.11	1.08 ± 0.07	1.18 ± 0.11	<0.05	<0.01	0.59
DNA methyltransferase 1	*Dnmt1*	CNS development, methylation	-1.85	<0.01	1.00 ± 0.06	1.04 ± 0.06	1.00 ± 0.08	1.03 ± 0.05	0.19	0.93	0.93
Minichromosome maintenance complex component 3	*Mcm3*	DNA strand elongation and replication	-1.87	<0.001	1.00 ± 0.07	0.97 ± 0.07	0.84 ± 0.06	0.90 ± 0.10	0.69	<0.01	0.15
dorsal inhibitory axon guidance protein	*Draxin*	Regulation of neuron projection development	-1.89	<0.001	1.00 ± 0.14	1.01 ± 0.09	0.69 ± 0.08	0.73 ± 0.06	0.59	<0.001	0.76
exportin 5	*Xpo5*	Transport of small and ds-RNA binding proteins	-1.90	<0.001	1.00 ± 0.71	0.98 ± 0.03	1.05 ± 0.08	1.14 ± 0.16	0.33	<0.05	0.17

*FDR* false discovery rate, *Nj* non-jaundiced control group, *jj* jaundiced group, *PT* phototherapy

#### Hyperbilirubinemia-specific transcriptomic effects

Of the 1701 DEGs associated with hyperbilirubinemia (Nj+no PT and Nj+PT vs. jj+no PT and jj+PT), 1294 were not associated with phototherapy (Nj+no PT and jj+no PT vs. Nj+PT and jj+PT). Of these, 895 DEGs were functionally annotated by IPA with 605 (68%) upregulated and 290 (32%) downregulated genes. The top biological processes affected by hyperbilirubinemia include increased remodeling of neurites, myelination, and abnormal morphology of glial cells (Fig. 2b).

#### Phototherapy-specific transcriptomic effects

Of the 3704 DEGs associated with phototherapy (Nj+no PT and jj+no PT vs. Nj+PT and jj+PT), 3297 were not associated with hyperbilirubinemia (Nj+no PT and Nj+PT vs. jj+no PT and jj+PT). Of these DEGs, only 2548 were analyzable by IPA with 1193 (47%) upregulated and 1355 (53%) downregulated genes. The top biological functions affected by phototherapy include activation of synaptic transmission and long-term potentiation (LTP) accompanied by inhibition of microtubule dynamics and neurogenesis (Fig. 2b).

#### Combined hyperbilirubinemia and phototherapy transcriptomic effects

Hyperbilirubinemia and phototherapy changed expression of 407 genes. 310 of these genes were annotated by IPA with 141 (45%) genes changed in opposing directions by hyperbilirubinemia and phototherapy, with 87 (21%) downregulated genes, and 123 (30%) upregulated genes by both conditions. These overlap gene expression changes indicate inhibition of neurite extension by hyperbilirubinemia with a partial recovery of such an effect by phototherapy. Phototherapy also induced gene expression changes indicating activation of cell viability (Fig. 2b and c).

#### qPCR validation of hippocampal transcriptome alterations in phototherapy groups

qPCR confirmed the expression pattern of all top upregulated and downregulated genes affected by phototherapy (*Ano3, Gabarapl2, Myo16, Vsnl1, Arhgef9, Rnfl6, Xpo5, Mcm3, Draxin)* except *Dnmt1*. *Myo16, Vsnl1*, and *Arhgef9* were also affected by hyperbilirubinemia. There was a hyperbilirubinemia*phototherapy interaction effect only for *Rnfl6* (Table 1).

#### Canonical signaling pathways and functions altered by hyperbilirubinemia or phototherapy

Comparative analysis of IPA-annotated pathways based on DEGs revealed two major clusters—oxidative phosphorylation and inflammatory signaling. Notably, oxidative phosphorylation signaling was increased by both hyperbilirubinemia and phototherapy. Hyperbilirubinemia resulted in activation of inflammatory signaling pathways, while phototherapy reduced activity of these pathways. While the jj groups (jj+no PT and jj+PT) did not have any functions that met the selection criteria (absolute z-score ≥ 2.0), the PT groups (Nj+PT and jj+PT) indicated greater neuronal dysgenesis and a reduced neuronal development and microtubule dynamics.

## Discussion

Utilizing the preterm-equivalent Gunn rat model of hyperbilirubinemia and phototherapy, this study demonstrates that mild hyperbilirubinemia, and to an even greater degree, phototherapy, induced transcriptomic changes in the developing rat hippocampus. The transcriptomic effects of bilirubin on the developing brain have been described in various models^29,39^ and for different brain regions,^27,28,30,31^ however, our results report gene expression changes for phototherapy, independent of the jaundice effect, which mapped onto canonical pathways and biological processes critical for neurogenesis, synaptic plasticity, and microtubule dynamics.

The Gunn rat has been used extensively for many decades to understand the pathophysiology of hyperbilirubinemia-induced brain injury.^40,41^ Some models use sulfadimethoxine (sulfa) injection to induce severe hyperbilirubinemia and bilirubin encephalopathy by displacing bilirubin from albumin and thereby increasing its transport into the brain.^18,19,42^ In clinical practice, particularly in the preterm population, it is common to blunt mild hyperbilirubinemia with phototherapy to prevent potential adverse effects of UCB.^4,43,44^ By delivering phototherapy between P4 and P6, which approximates human preterm infants from a brain development perspective,^33,35^ we assessed the short-term transcriptomic effects of phototherapy on the hippocampus, a brain region vulnerable in neonatal hyperbilirubinemia. While the effects of naturally occurring hyperbilirubinemia,^28,45,46^ phototherapy,^22–25^ and preterm equivalent timelines^18,28,46^ have been studied previously in the Gunn rat, most of these studies have focused on the cerebellum.^18,23,28,45^ Our model highlights hippocampal transcriptomic effects in the context of hyperbilirubinemia and its treatment with phototherapy during a preterm equivalent developmental period.

Our study confirms previous work including transcriptomic studies demonstrating that hyperbilirubinemia impacts the developing brain through the dysregulation of genes regulating neurite remodeling,^39^ myelination,^28^ and neuroinflammation.^29^ Hippocampal cell culture experiments demonstrate that UCB negatively affects neurite arborization and neuron connectivity.^47^ While UCB’s primary target are neurons with their myelin-rich cell membranes,^43^ our work corroborates UCB’s effect on glial cells as well. Glial cells,^48^ including astrocytes,^49,50^ microglia,^51,52^ and oligodendrocytes,^53–55^ can be impacted by UCB, resulting in dysregulated inflammatory responses and impaired myelination. Our study using in vivo ^1^H MR Spectroscopy in a preterm Gunn rat model demonstrated impaired myelination and gliosis in the cerebellum.^38^ The present study adds to this body of work, highlighting that UCB alters the function of aforementioned cell types in the hippocampus.

Of particular interest to this study was specific effect of phototherapy on the hippocampal transcriptome. Phototherapy altered expression of over two-times as many genes as hyperbilirubinemia, a pattern that mirrors our prior study of plasma metabolites in human preterm infants with hyperbilirubinemia that showed greater metabolite changes with phototherapy exposure.^7^ Importantly, the major effects of phototherapy-altered hippocampal gene expression center on neuronal development, synaptic plasticity and microtubule remodeling, suggesting that phototherapy exposure alone can exert genomic modifications in metabolically active brain structure (e.g., developing hippocampus).

Previous studies demonstrate that neurogenesis, including microtubule dynamics that support brain development, and synaptic transmission, including calcium-mediated cell signaling, are affected by hyperbilirubinemia^27,31,38,39,47,56^ To our knowledge, this is the first study to identify an independent effect of phototherapy on these processes. Significant changes to hippocampal gene expression in our model included *Vsnl1*, *Rnf6*, and *Draxin*, which are involved in regulation of neurite remodeling, were altered in the phototherapy groups but not in the jaundice-no jaundice group comparison (Table 1). Genes involved in cell signaling and synaptogenesis including *Ano3* and *Arhgef9* were significantly upregulated in the phototherapy groups with no differences in expression between jaundiced and non-jaundiced groups (Table 1). Of note, *Arhgef9* is an X-linked gene that encodes for collybistin, a protein involved in regulating postsynaptic density.^57^ Its variants have been implicated in epileptic phenotypes in the literature.^57–59^ Given phototherapy’s association with increased risk of seizures in males,^8^ how phototherapy interacts with *Arhgef9* and its associated pathways warrant further exploration. Overall, these data are consistent with the alterations in important neuronal functions, including membrane lipid metabolism, oligodendrocyte differentiation, and creatine biosynthesis with phototherapy in the plasma of preterm infants with hyperbilirubinemia we recently reported.^7^

Our study focused on the developing hippocampus as this brain region is vulnerable to the consequences of hyperbilirubinemia. The hippocampus is important for cognition and learning and memory, and preterm infants are at an overall risk for long-term hippocampal-mediated effects.^26,60^ Insults during this stage of hippocampal development can have long-term impact on hippocampal structure and function.^61,62^ While the majority of preterm infants receive phototherapy for the treatment of neonatal hyperbilirubinemia,^7^ we currently lack evidence-based treatment guidelines for treatment in this population, which makes this and similar studies even more relevant. Knowledge of brain region-specific effects of bilirubin and various timelines and irradiance doses of phototherapy would inform translational studies to further investigate the effects of treatment in human neonates.

Limitations of these data include an absence of neurobehavioral outcomes as well as long-term gene expression changes. The purpose of this study was discovery-based analysis to pinpoint molecular networks and lay a groundwork for future investigations. Next steps will determine the biological relevance of the identified molecular pathways as well as evaluate whether the short-term gene expression changes we identified persist long-term. Our study provides the model for phototherapy in a preterm Gunn rat model demonstrating hippocampal effects. We demonstrated tissue-specific effects in the hippocampus, but whether these effects are localized to the hippocampus or also present in other brain regions (for example, the cerebellum)^18,23,28,45^ or reflect systemic effects were not tested.

## Conclusions

This study demonstrates that hyperbilirubinemia and to a greater degree its treatment with phototherapy alters the transcriptome of the preterm hippocampus. Phototherapy lowered serum bilirubin levels and altered genes involved in neurogenesis, synaptic plasticity, and microtubule dynamics.

## Supplementary information


Supplementary Material
Supplementary Table S1
Supplementary Table S2


## Data Availability

The RNAseq datasets generated during the current study are available in GEO repository (acquisition number GSE189509), and all other data are available upon reasonable request to the corresponding author.
