# Supplementary material for: Hyperbilirubinemia and phototherapy differentially alter hippocampal transcriptome in the preterm Gunn rat model
Source: Pediatr Res. 2025 Dec 22;100(1):439–45. doi: 10.1038/s41390-025-04700-y (PMC13524819; doi:10.1038/s41390-025-04700-y)
Supplement: Supplementary file 1 [file 41390_2025_4700_MOESM2_ESM.pdf]

Supplementary Table S1: qPCR Probe Identification Information

Gene name	Gene Symbol	GenBank mRNA or RefSeq Accession No.	TaqMan Assay ID
anoctamin 3	*Ano3*	XM_230381.8	Rn01515473_m1
GABA(A) receptor-associated protein like 2	*Gabarapl2*	NM_022706.2	Rn01426783_g1
myosin XVI	*Myo16*	NM_138893.1	Rn00594181_m1
visinin-like 1	*Vsnl1*	NM_012686.2	Rn01644700_m1
DNA (cytosine-5-)-methyltransferase 1	*Dnmt1*	NM_053354.3	Rn00709664_m1
Cdc42 guanine nucleotide exchange factor (GEF) 9	*Arhgef9*	NM_023957.1	Rn00576661_m1
exportin 5	*Xpo5*	NM_001108789.3	Rn01512222_m1
ring finger protein (C3H2C3 type) 6	*Rnf6*	NM_001107118.1	Rn01453885_m1
minichromosome maintenance complex component 3	*Mcm3*	NM_001191805.2	Rn01511840_m1
dorsal inhibitory axon guidance protein	*Draxin*	XM_006239423.2	Rn01520059_m1
ribosomal protein S18	*Rps18*	NM_213557.1	Rn01428913_gH
