# Supplementary material for: Hyperbilirubinemia and phototherapy differentially alter hippocampal transcriptome in the preterm Gunn rat model
Source: Pediatr Res. 2025 Dec 22;100(1):439–45. doi: 10.1038/s41390-025-04700-y (PMC13524819; doi:10.1038/s41390-025-04700-y)
Supplement: Supplementary file 2 [file 41390_2025_4700_MOESM3_ESM.pdf]

Supplementary Table S2: Overall interaction and pair-wise comparisons by ANOVA for hippocampal transcript expression by RNA sequencing. The top ten genes identified for each group comparison are listed with the associated fold change, all with a $p < 0.001$.

Group Comparisons														
	Overall Interaction		Nj+PT vs. Nj+no PT		jj+PT vs. jj+no PT		Nj+no PT vs. jj+no PT		Nj+PT vs. jj+PT		jj+PT vs. Nj+no PT		Nj+PT vs. jj+no PT	
	Number of Genes with p<0.05	710	Number of Genes with p<0.05	2909	Number of Genes with p<0.05	2218	Number of Genes with p<0.05	1204	Number of Genes with p<0.05	1192	Number of Genes with p<0.05	2795	Number of Genes with p<0.05	3712
**Top 10 Genes Identified**	**p-value**	**FDR**	**Gene Symbol**	**Fold Change**	**Gene Symbol**	**Fold Change**	**Gene Symbol**	**Fold Change**	**Gene Symbol**	**Fold Change**	**Gene Symbol**	**Fold Change**	**Gene Symbol**	**Fold Change**
Ntrk3	6.2969 e-5	0.93	*Ttll7*	1.87	*Nedd4l*	-2.14	*Kdm8*	-2.10	*Fbxl21*	-2.18	*Ttll7*	1.56	*Ninl*	-2.52
Gabara6-AS1	0.0001	0.93	*Sf3b3*	-1.91	*Cachd1*	-1.68	*Ciart*	-2.01	*RT1-A2*	-1.82	*Sf3b3*	-1.83	*Ttll7*	1.45
Myh6	0.0002	0.93	*Eps15*	1.83	*Apcdd1*	-1.70	*Ntrk3*	-1.18	*Tra2a*	1.51	*Nedd4l*	-2.24	*Cachd1*	-1.73
Pyy	0.0002	0.93	*Uba1*	-2.10	*Adamts7*	-1.99	*Pdzrn3*	-1.63	*Pptc7*	-1.46	*Uba1*	-2.05	*Sptssb*	2.45
Coa6	0.0004	0.93	*Prag1*	-2.46	*Sf3b3*	-1.51	*Anp32a*	2.17	*Uba2*	1.81	*Itpka*	1.79	*Eps15*	1.55
Hmbox1	0.0005	0.93	*Fadd*	-1.58	*Tnc*	-1.77	*Itgal*	-2.01	*Tmem8a*	-1.81	*Rnf6*	2.09	*Ufsp2*	2.30
Lipf	0.0005	0.93	*Zbtb12*	-2.15	*Zfp609*	-1.64	*Kcnq3*	-1.42	*Eif4e3*	-1.46	*Limch1*	-2.37	*Sf3b3*	-1.59
Tekt3	0.0006	0.93	*Xpo5*	-2.26	*Ptpn9*	-2.22	*Adgre1*	-1.81	*Cyp4f17*	-2.00	*Eps15*	1.60	*Tnc*	-1.88
Caln1	0.0008	0.93	*Lpcat1*	-2.15	*Rcbtb2*	-2.33	*Polr2h*	1.97	*Klf13*	-1.21	*Klf13*	1.70	*Fadd*	-1.46
Foxn3	0.0008	0.93	*Cachd1*	-1.65	AABR07027575.1	-1.82	*Ncf1*	-2.05	*Chp2*	-1.28	*Ubap2l*	-2.14	*Flnc*	-2.27
